# Localization of Sensorimotor Cortex Using Navigated Transcranial Magnetic Stimulation and Magnetoencephalography

**DOI:** 10.1007/s10548-019-00716-w

**Published:** 2019-05-15

**Authors:** Minna Pitkänen, Shogo Yazawa, Katja Airaksinen, Pantelis Lioumis, Jussi Nurminen, Eero Pekkonen, Jyrki P. Mäkelä

**Affiliations:** 1 Department of Neuroscience and Biomedical Engineering, Aalto University School of Science, Espoo, Finland; 2 Department of Clinical Neurophysiology, Kuopio University Hospital, Kuopio, Finland; 3 A. I. Virtanen Institute for Molecular Sciences, University of Eastern Finland, P. O. Box 1627, 70211 Kuopio, Finland; 4 Department of Systems Neuroscience, Sapporo Medical University, Sapporo, Japan; 5 BioMag Laboratory, HUS Medical Imaging Center, Helsinki University Hospital and University of Helsinki, Helsinki, Finland; 6 Department of Neurology, Helsinki University Hospital, Helsinki, Finland; 7 Department of Clinical Neurosciences (Neurology), University of Helsinki, Helsinki, Finland

**Keywords:** Navigated transcranial magnetic stimulation, Magnetoencephalography, Motor evoked potential, Corticomuscular coherence, Corticokinematic coherence, Somatosensory evoked field

## Abstract

The mapping of the sensorimotor cortex gives information about the cortical motor and sensory functions. Typical mapping methods are navigated transcranial magnetic stimulation (TMS) and magnetoencephalography (MEG). The differences between these mapping methods are, however, not fully known. TMS center of gravities (CoGs), MEG somatosensory evoked fields (SEFs), corticomuscular coherence (CMC), and corticokinematic coherence (CKC) were mapped in ten healthy adults. TMS mapping was performed for first dorsal interosseous (FDI) and extensor carpi radialis (ECR) muscles. SEFs were induced by tactile stimulation of the index finger. CMC and CKC were determined as the coherence between MEG signals and the electromyography or accelerometer signals, respectively, during voluntary muscle activity. CMC was mapped during the activation of FDI and ECR muscles separately, whereas CKC was measured during the waving of the index finger at a rate of 3–4 Hz. The maximum CMC was found at beta frequency range, whereas maximum CKC was found at the movement frequency. The mean Euclidean distances between different localizations were within 20 mm. The smallest distance was found between TMS FDI and TMS ECR CoGs and longest between CMC FDI and CMC ECR sites. TMS-inferred localizations (CoGs) were less variable across participants than MEG-inferred localizations (CMC, CKC). On average, SEF locations were 8 mm lateral to the TMS CoGs (*p *< 0.01). No differences between hemispheres were found. Based on the results, TMS appears to be more viable than MEG in locating motor cortical areas.

## Introduction

Sensorimotor cortex (SM1) is often mapped prior to brain surgery to plan and optimize the operation. The mapping can be performed using, e.g., navigated transcranial magnetic stimulation (TMS) (Paiva et al. 2012; Picht et al. 2011; Tarapore et al. 2012; Vitikainen et al. 2009, 2013) or magnetoencephalography (MEG) (Mäkelä et al. 2001; Tarapore et al. 2012).

TMS is a non-invasive method to investigate cortical function (Barker et al. 1985). When TMS is applied on the motor cortex, it may induce motor evoked potentials (MEPs) measured with electromyography (EMG). MEPs reflect the excitatory motor functions of the cortex and the spinal cord and are often used in the cortical mapping but also in the diagnosis and follow-up of some diseases. In TMS, cortical motor representations are determined by applying single magnetic pulses within the precentral gyrus and recording MEPs. The mapping is continued to the surrounding areas until no MEPs are evoked. Often, the center of gravity (CoG) is calculated to determine the amplitude-weighted center of the representation (Wassermann et al. 1992). CoG can be utilized, for example, to investigate the plastic changes in the motor areas during rehabilitation after brain trauma. The spatial accuracy of the electric field navigated TMS is better than 6 mm (Hannula and Ilmoniemi 2017; Schmidt et al. 2015).

MEG is also a non-invasive method to measure brain activity. It measures the magnetic fields generated by the currents in the brain tissue with super-conducting sensors outside the head (Hämäläinen et al. 1993). MEG has temporal resolution of milliseconds and spatial resolution of few millimeters in an optimal situation (Hämäläinen et al. 1993). Previous MEG studies have used, for instance, somatosensory evoked fields (SEFs) or event-related desynchronization (ERD) to determine the SM1 (Mäkelä et al. 2001; Tarapore et al. 2012). Alternative approaches to MEG motor mapping are cortico-muscular coherence (CMC) and cortico-kinematic coherence (CKC) (Bourguignon et al. 2011, 2013; Conway et al. 1995; Jerbi et al. 2007; Salenius et al. 1997). Coherence is a measure of the linear dependence between two signals and reflects their amplitude and phase correlation within a frequency band. The coherence values range between 0 and 1: the coherence of two identical signals equals 1, whereas for the signals that are not related at all, the coherence equals 0. CMC represents the interaction between cortical processing measured with MEG or electroencephalography (EEG) and simultaneous isometric muscle contractions measured with EMG (Conway et al. 1995). CKC refers to the coherence between MEG or EEG signal and the kinematics of repetitive movement measured with accelerometer (ACC) (Bourguignon et al. 2011). CMC is often detected at the beta frequency rate (around 15–30 Hz) (Conway et al. 1995; Johnson et al. 2011; Kilner et al. 1999; Salenius et al. 1997), whereas CKC is observed at the movement frequency and its first harmonic component (Bourguignon et al. 2011). The cortical location showing the highest coherence at these frequency ranges is assumed to be the site of the motor representation (Salenius et al. 1997). In physiological studies of the motor system, the EMG used in CMC is typically recorded from small hand muscles (Salenius et al. 1997), whereas studies of pathophysiology of, e.g., Parkinson’s disease have preferred wrist extensor muscle EMG (Airaksinen et al. 2015b).

Previously, TMS motor and MEG somatosensory and motor ERD mappings have been validated with invasive direct cortical stimulation (DCS) (Mäkelä et al. 2001; Paiva et al. 2012; Picht et al. 2011; Tarapore et al. 2012). Comparison of MEG CMC mapping with DCS has been reported in two patients (Mäkelä et al. 2001, 2013); MEG CKC mappings have not, to our knowledge, been compared with DCS. Integration of the TMS mapping data into the clinical hospital databases is relatively straightforward (Mäkelä et al. 2015), whereas the analysis and incorporation of the MEG data are somewhat more elaborate. Direct comparison between MEG and TMS mappings has been conducted mainly in patients. In tumor patients, TMS hotspot, i.e., the cortical location of the maximum MEP, has been observed to correlate well with ERD and DCS locations (Tarapore et al. 2012). In an epilepsy patient, TMS and CMC locations were similar, but DCS results were different (Mäkelä et al. 2013). Also SEFs have been mapped to locate the SM1 (Morioka et al. 1995; Mäkelä et al. 2001; Vitikainen et al. 2009). To our knowledge, only one study has compared MEG and TMS motor mappings in a healthy subject (Ruohonen et al. 1996). The study involved only one subject and mapped MEG motor readiness fields.

DCS is the gold standard for brain mapping but due to its invasiveness, it can be performed only in eligible patients during surgery. Therefore, in the present study of healthy subjects, relative differences between TMS CoGs and MEG CMC, CKC, and SEF locations were investigated. The results will provide new insights into differences between the methods.

## Methods

### Measurements

Ten healthy volunteers participated in the study (age 22–58 years, 6 males, 9 right-handed). The subjects did not have any contraindications to TMS, MEG, or magnetic resonance imaging (MRI). Written informed consent was received from all subjects, and the study was approved by the ethical committee of the Hospital District of Helsinki and Uusimaa (Finland) in accordance with the declaration of Helsinki. The subjects had first MEG mapping followed immediately with TMS mapping. In both mappings, the surface EMG electrodes were the same and attached to the skin above first dorsal interosseous (FDI) and extensor carpi radialis (ECR) muscles. Both hemispheres were mapped. Vertical and horizontal electro-oculography eye movements were recorded during MEG.

### MEG

MEG was measured with a 306-channel neuromagnetometer (Elekta Neuromag, Elekta Oy, Helsinki, Finland). Mappings were performed in the following order: (1) SEF during tactile stimulation, (2) CKC during the waving of the index finger, (3) CMC during wrist dorsiflexion (CMC ECR), and (4) CMC during squeezing a ball (CMC FDI).

SEFs were elicited by tactile stimulation (duration 141 ms, peak 50 ms) using a balloon diaphragm driven by compressed air once in every 1 s alternatively to the left and right index finger. During CKC measurement, the subjects were asked to wave their index finger at a rate of 3–4 Hz for 1 min. The activation was repeated three times for both hands and the interval between the trials was 20 s. The hands were held in a neutral position between pronation and supination to avoid tactile sensation in the fingertips. The kinematics were recorded with ACC device (ADXL335 iMEMS Accelerometer, Analog Devices Inc., Norwood, MA, USA), which was attached to the nails of the index fingers. The acceleration was measured in three orthogonal directions. A small screen prevented the subjects from seeing the moving hand to avoid the movement-related activation of the visual system in the brain.

In the CMC measurement, two tasks were performed. For CMC of FDI, the subjects squeezed a squash ball (soft, two points). For CMC of ECR, the subjects were instructed to extend their wrist for 1 min at a time. In a preliminary trial before the actual task, the subjects extended their wrist with the maximal force for comparison with the task contraction force. The aim was to produce a sustained isometric contraction by using submaximal force. The tasks were repeated five times with a pause of 20 s between each trial. At the end of the MEG measurements, 120-s empty-room data were recorded.

### TMS

Navigated TMS mapping was done immediately after the MEG recordings with a biphasic waveform and a figure-of-eight coil (version 4.3. Nexstim Plc. Helsinki, Finland). First, the precentral gyrus and its surroundings were roughly mapped to find the hotspot location. Then the resting motor threshold (rMT) was determined in that location using the Rossini–Rothwell method with 10 pulses (Rossini et al. 2015; Rothwell et al. 1999). A more precise mapping was started from the hotspot and continued to the surrounding areas until no MEPs were induced. MEPs equal to or greater than 50 µV in peak-to-peak amplitude were accepted. Both hemispheres and muscles were mapped separately in a randomized order at the stimulation intensity of 105–110% rMT. The direction of the electric field was kept perpendicular to the central sulcus. EMG signal was visually inspected during mapping to ensure that the recorded muscles were resting.

### Data analysis

#### MEG

To suppress external artifacts, the spatiotemporal signal space separation was applied with an 8-s time window and a subspace correlation limit of 0.9 (Airaksinen et al. 2015a; Medvedovsky et al. 2009; Taulu and Simola 2006) by the MaxFilter software (Elekta Oy). MaxFilter was also used to compensate head movements, which were continuously tracked with respect to the MEG helmet.

The MRI segmentation and reconstruction of cortical surfaces, the coregistration of MEG with individual MRI of each subject, the computation of the forward solution, and calculation of the inverse problem were conducted with FreeSurfer (version 5.3, Martinos Center for Biomedical Imaging, MA, USA) and MNE software (version 2.7.4, Martinos Center for Biomedical Imaging, Gramfort et al. 2014). The source estimates were computed and other analyses were conducted with MNE–Python (Gramfort et al. 2013, 2014). Only gradiometer signals were considered in the analysis and the eye movement artifacts were removed with the independent component analysis.

In the SEF analysis, the raw data were filtered at 1–195 Hz and notch filtered at 50 Hz and its harmonics. Epochs were determined from − 0.1 to + 0.2 s with respect to the stimulation onset (Bourguignon et al. 2013). The number of averaged epochs was 100–150. Dipole fitting for averaged epochs was done with 0.1 ms intervals around the visually determined peaks in sensor signals (Nevalainen et al. 2012). Only the dipoles with goodness-of-fit equal or greater than 70% were accepted and the dipole with the greatest dipole moment was chosen to represent the location of SM1 (Nevalainen et al. 2012).

In CMC and CKC analysis, the raw data were filtered at 1–100 Hz and notch filtered at 50 Hz and its harmonics. The muscle activation times were checked from EMG or ACC signals and the first 5 s and the last 3 s of every trial were discarded (Pohja et al. 2005). The source estimates constrained at SM1 contralateral to the activated hand were calculated using minimum-norm estimation (MNE) (Hämäläinen and Ilmoniemi 1994). CMC was calculated between the unrectified EMG signal and MEG sources using a discrete Fourier transform with 50% overlapping 1024-ms Hanning windows at frequencies 15–30 Hz (Airaksinen et al. 2015a). CKC was calculated between the norm of the ACC signal and MEG sources using a discrete Fourier transform with 80% overlapping 2-s Hanning windows at frequencies 2–10 Hz (Bourguignon et al. 2016). Some subjects showed two or more CKC peaks and the first clear peak was chosen for further analysis. The cortical location of the maximum coherence was chosen to represent SM1. The coherence was considered significant if it exceeded the maximum of the time-shifted coherence, which was calculated by shifting the EMG or ACC signal by 3 s (Airaksinen et al. 2015a, b).

#### TMS

In the preprocessing of TMS data, MEPs lower than 50 µV in peak-to-peak amplitude were treated as non-responses. EMG signal was visually checked for muscle activity and the trials during muscle tension were rejected from analysis. CoGs were calculated as the MEP amplitude-weighted sum of cortical locations. Matlab (R2014b, MathWorks Inc., MA, USA) was used in the TMS data analysis.

### Statistical analysis

All coordinates were analyzed in MNI305 space (Collins et al. 1994; Evans et al. 1992) (x = medial–lateral, y = posterior–anterior, z = inferior–superior) and the Euclidean distances between the locations were calculated. Statistical calculations were performed with SPSS (IBM SPSS Statistics, version 22, NY, USA). The significance level was set at *p *< 0.05. The absolute values of the coordinates in the medial–lateral direction were used in the statistical analysis, which allows comparison between the hemispheres.

## Results

**Fig. 1:**
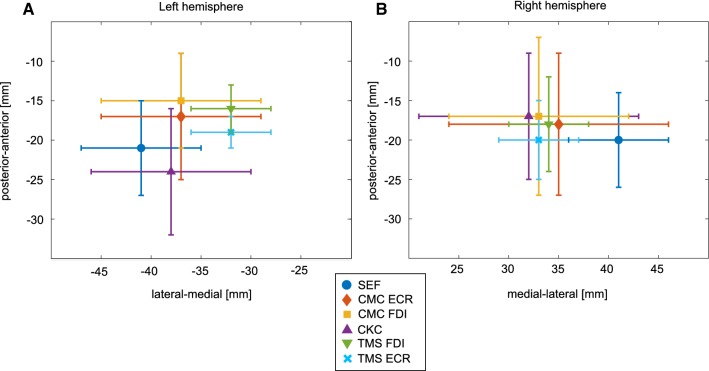
The x- and y-coordinates of the localizations in MNI space in **a** left and **b** right hemisphere (mean ± standard deviation)

**Fig. 2:**
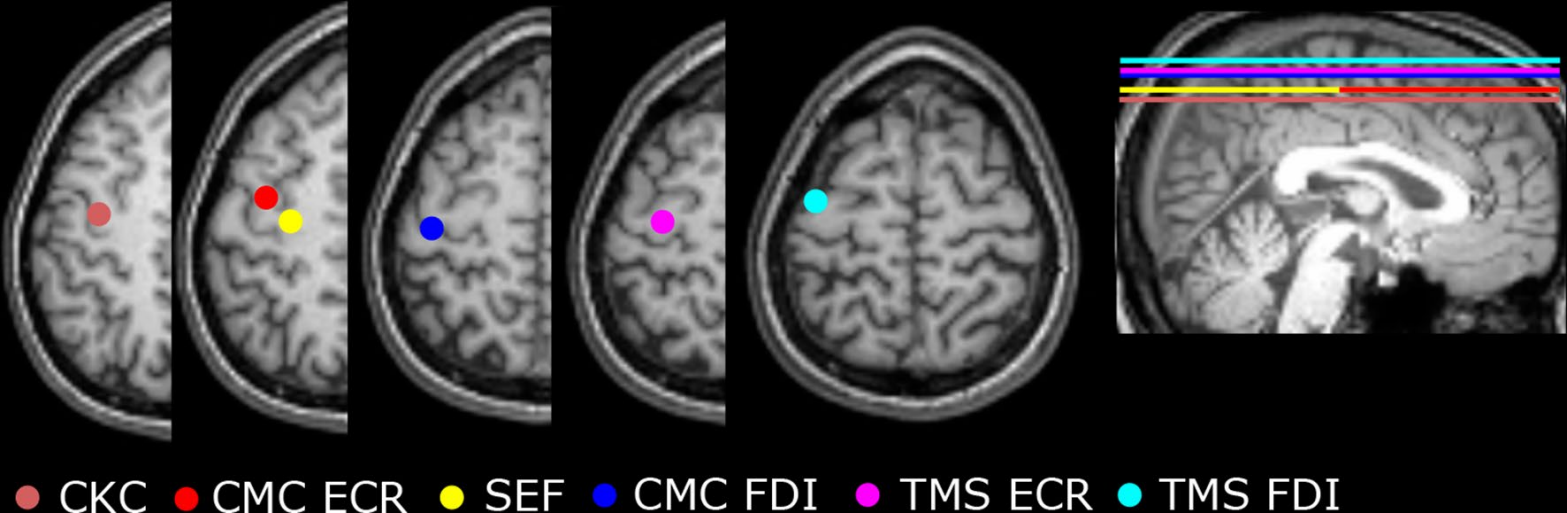
The cortical localizations of the right hand mappings in one subject

SEF responses were observed in all subjects. The location of the SEF was in the central sulcus approximately in 50% of cases and on the postcentral gyrus in 30% of the cases. The location of the maximum CMC was on the precentral gyrus in approximately 56% of the mappings, whereas the location of maximum CKC was on the precentral gyrus in approximately 25% of the cases. The CMC FDI result of one subject was excluded from the study because the frequency (29 Hz) and cortical location (in lateral parts of the postcentral gyrus) of the maximum coherence differed greatly from the other cases and the coherence was only slightly above the significance level. The coherence in this case was insignificant at other frequencies. TMS located FDI and ECR CoGs on the precentral gyrus in all subjects. The average x- and y-coordinates of different mappings are shown in Fig. 1 and the localizations in one subject are shown in Fig. 2.

**Fig. 3:**
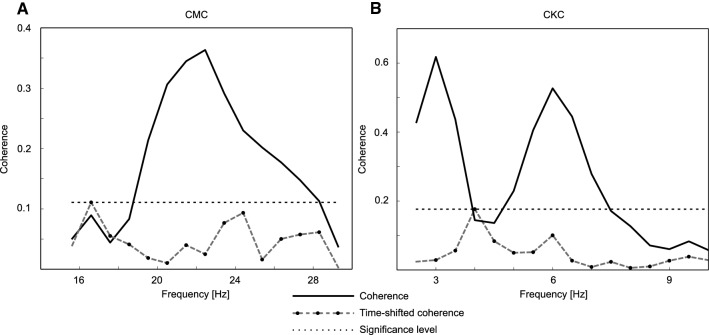
Examples of **a** cortico-muscular coherence (CMC), **b** cortico-kinematic coherence (CKC), and the time-shifted CMC/CKC from the cortical source producing the maximum coherence in each case in one subject. The significance level was chosen based on the maximum of the time-shifted coherence

The means of the maximum coherence values and the corresponding frequencies are presented in Table 1 and examples of CMC and CKC as a function of frequency are shown in Fig. 3. The mean Euclidean distances between the localizations are presented in Table 2. On average, the TMS CoGs of FDI and ECR muscles had the shortest distance (5 ± 3 mm, mean ± standard deviation), whereas CMC of FDI and ECR had the longest distance (20 ± 13 mm). Even though the distances varied the mean coordinates of different mappings were closely located (Fig. 1).

**Table 1:** Maximum coherences and the frequencies (mean ± standard deviation)

	Coherence	Frequency (Hz)
CMC FDI	0.2 ± 0.1	21 ± 3
CMC ECR	0.3 ± 0.1	23 ± 4
CKC	0.6 ± 0.2	3 ± 1

**Table 2:** The Euclidean distances [mm] between the localizations (mean ± standard deviation)

	CMC FDI	CMC ECR	CKC	CoG FDI	CoG ECR
SEF	18 ± 8	18 ± 9	16 ± 7	14 ± 4	13 ± 5
CMC FDI		20 ± 13	19 ± 10	14 ± 10	14 ± 8
CMC ECR			16 ± 13	14 ± 9	14 ± 8
CKC				16 ± 4	15 ± 5
CoG FDI					5 ± 3

The Linear Mixed Model analysis indicated that the localizations differed based on the mapping method in x- (*F *= 3.47, *p *= 0.006) and z-directions (*F *= 2.91, *p *= 0.017) but not in y-direction (*F *= 1.53, *p *= 0.187). Pairwise comparison indicated that the mean SEF location was 8 mm lateral to the TMS ECR and FDI CoGs (*p *= 0.007 and *p *= 0.009, respectively). In addition, the mean z-coordinate of the SEF location was approximately 8 mm smaller than that of the CKC location (*p *= 0.011) indicating deeper SEF source. The mappings did not differ between hemispheres in x- (*F *= 0.70, *p *= 0.404), y- (*F *= 0.08, *p *= 0.782), and z-directions (*F *= 0.50, *p *= 0.481).

## Discussion

TMS and MEG are both used in motor mapping which is useful in presurgical evaluations and in the investigation of cortical reorganization. Therefore, in this study, the congruence between these mapping methods was evaluated. In the MEG mappings, CMC, CKC and SEF locations were calculated, whereas TMS locations were based on the computation of CoGs. Previously, MEG and TMS mappings have been compared by using SEFs, ERD, and CMC mainly in patients (Mäkelä et al. 2013; Tarapore et al. 2012; Vitikainen et al. 2009). These studies indicated that MEG and TMS motor maps are closely located and often correlated well with DCS. To our knowledge, this is the first study comparing MEG CMC, CKC, SEF, and TMS CoG mappings in healthy individuals.

All mappings localized SM1 on average within 20 mm. On average, the shortest distances were between two TMS mappings and the longest distances were between two CMC mappings. The TMS locations were found to be more medial than the SEF sites. The average distance between the TMS and MEG localizations (approximately 14 mm) is comparable to or slightly larger than previously found distances between TMS and functional magnetic resonance imaging (Diekhoff et al. 2011; Herwig et al. 2002; Kallioniemi et al. 2016; Lotze et al. 2003). However, an earlier comparison between TMS hotspots and MEG ERD locations revealed shorter distances than observed in the present study (Tarapore et al. 2012). The differences in the results may be due to different analysis procedures; the source estimation method, for example, may influence the results. The earlier study investigated the distances between TMS hotspots and MEG ERD locations (Tarapore et al. 2012), whereas in the current study, TMS CoG and MEG CMC, CKC, and SEF sites were compared. In addition, the previous study projected the MEG locations to the cortical surface (Tarapore et al. 2012), which likely shifts the MEG locations closer to the TMS sites. They also compared DCS results with TMS and MEG and concluded that on average the distance between TMS hotspots and DCS sites was shorter than the distance between MEG and DCS locations (Tarapore et al. 2012).

The differences in the mappings may be due to the characteristics of the methods. TMS mapping is based on the induced involuntary activation of the muscles, whereas MEG CMC and CKC are measured during voluntary activation; SEFs reflect the tactile processing. Thus, the mapped neuronal populations may be different. Moreover, MEG is preferentially sensitive to tangential currents and, hence, it locates activation typically in the sulci (Hämäläinen et al. 1993). The direction of the neurons affects the TMS outcome as well, and therefore TMS is typically applied so that the maximal electric field is perpendicular to the sulci in the posterior–anterior direction. TMS-induced activation is assumed to occur at axons that bend or terminate within the electric field or along axons with the greatest electric field gradient (Silva et al. 2008; Tranchina and Nicholson 1986), whereas MEG signal likely originates from the postsynaptic activity (Hämäläinen et al. 1993).

Neither MEG nor TMS can accurately reveal deep sources. The depth of TMS is more artificial because the peeling depth is set by the researcher at the beginning of the measurement and the TMS locations are on that surface. Moreover, in the MEG mappings, different tasks may have influenced the results. For example, SEFs are typically located in the central sulcus or postcentral gyrus representing the somatosensory instead of motor functions. In MEG measurements, various muscles may be activated simultaneously and somatosensory function may have an effect on the localizations in the CMC FDI task in which the fingers touched the ball, and due to proprioceptive movement-related feedback during CKC (Bourguignon et al. 2012; Piitulainen et al. 2013).

CKC appears to be more sensitive to SM1 mapping than CMC (Bourguignon et al. 2011, 2013). In the present study, CKC values were higher, which supports this assumption. Previous studies suggested that CKC reflects movement-related somatosensory proprioceptive afferent input to the SM1 (Piitulainen et al. 2013).

CMC has been observed to be lacking in some subjects and its magnitude varies between subjects (Pohja et al. 2005). The reason for this is unclear. Matsuya et al. hypothesized that the inter-subject variability may be associated with the inhibitory interneuron networks at the cortical and spinal levels (Matsuya et al. 2017). In the present study, the CMC and CKC were found in all subjects. In one subject, however, the frequency and cortical location of the maximum CMC FDI deviated strongly from those of the other subjects and was excluded from the analysis. Moreover, in five subjects the CMC FDI of a hand and in one subject CMC ECR of the right hand was only slightly above the significance level. On average, CMC ECR values were higher than CMC FDI values. Therefore, CMC ECR appears to be a more robust method than CMC FDI, which favors the use of ECR recordings in clinical studies. Previous studies have commonly calculated coherence at the sensor level, whereas in this study, it was calculated at the source level. This may have influenced the level of CMC and CKC.

The CoGs were chosen to represent the TMS mappings in this study as they are more reliable than the hotspots (Weiss et al. 2013). The CoGs consider the whole map area but may not, however, be optimal locations for stimulation. This may have affected the present results. One stimulation trial was given at each cortical location to reduce the measurement duration, which was considered reliable based on previous studies (Herwig et al. 2002; Pitkänen et al. 2015). In one previous study, the maps of two single-pulse measurements did not differ suggesting that one pulse at each cortical location is sufficient (Pitkänen et al. 2015). The single-pulse per location approach may influence the hotspots but as CoGs are calculated based on several MEPs, the effect is likely smaller. In the present study, two TMS mappings resulted in closely located CoGs indicating reproducible methods.

A limitation of this study is that the effect of handedness could not be studied in detail, because only one of the subjects was left handed. Previously, it has been suggested that the handedness affects the motor representations mapped with TMS (Nicolini et al. 2019) and thus it might have influenced the results of the current study.

Based on the results and due to the more straightforward analysis of TMS data, TMS mapping may be a better choice than MEG for fast functional mapping, as it enables a fluent integration of the mapping results into clinical practice (Mäkelä et al. 2015). However, because we could not map functional areas of healthy volunteers with DCS due to its invasiveness, we cannot draw conclusions about the accuracy of the TMS and MEG mappings. All TMS CoGs were on the precentral gyrus, which is usually the location of motor function, whereas MEG sites were scattered to a wider area. When comparing TMS and MEG motor sites with MEG SEF location, the average location of SEF was more posterior and lateral than most of the TMS and MEG based landmarks. The mean Euclidean distances were shorter between TMS and SEF than between MEG and SEF, which could imply that the TMS CoG mapping is a feasible method in locating somatosensory cortex. However, based on the statistical analysis, a significant difference between TMS CoGs and SEF locations was found in medial–lateral direction. Patients who suffer from motor deficits or have poor cooperation may have difficulties in performing the motor tasks mandatory in MEG motor mapping. Further, SEFs might not be elicited in patients suffering from peripheral polyneuropathy. Finally, TMS is more widely available than MEG due to the high cost and infrastructure requirements of MEG.
